# Role of oral pathogens in the pathogenesis of intracranial aneurysm: review of existing evidence and potential mechanisms

**DOI:** 10.1007/s10143-020-01253-y

**Published:** 2020-02-07

**Authors:** Joona Hallikainen, Sara Keränen, Jarno Savolainen, Matti Närhi, Anna Liisa Suominen, Pekka Ylöstalo, Jari Kellokoski, Mikko Pyysalo, Pirkko Pussinen, Tuomas Rauramaa, Juhana Frösen

**Affiliations:** 1 Institute of Dentistry, University of Eastern Finland, Kuopio, Finland; 2 Department of Oral and Maxillofacial Diseases, Kuopio University Hospital, Puijonlaaksontie 2, 70210 Kuopio, Finland; 3 Hemorrhagic Brain Pathology Research Group, Kuopio University Hospital, Kuopio, Finland; 4 Department of Biomedicine, University of Eastern Finland, Kuopio, Finland; 5 Public Health Evaluation and Projection Unit, National Institute for Health and Welfare, Helsinki, Finland; 6 Medical Research Center, Oulu University Hospital and University of Oulu, Oulu, Finland; 7 Hemorrhagic Brain Pathology Research Group, Tampere University Hospital and University of Tampere, Tampere, Finland; 8 Oral and Maxillofacial Diseases, University of Helsinki and Helsinki University Hospital, Helsinki, Finland; 9 Department of Pathology, Kuopio University Hospital and University of Eastern Finland, Kuopio, Finland; 10 Department of Neurosurgery, Tampere University Hospital and University of Tampere, Tampere, Finland

**Keywords:** Intracranial aneurysm, Periodontitis, Pathogens, Oral diseases, Subarachnoidal hemorrhage

## Abstract

Degeneration of intracranial aneurysm wall is under active research and recent studies indicate an increased risk of rupture of intracranial aneurysm among patients with periodontal diseases. In addition, oral bacterial DNA has been identified from wall samples of ruptured and unruptured aneurysms. These novel findings led us to evaluate if oral diseases could predispose to pathological changes seen on intracranial aneurysm walls eventually leading to subarachnoid hemorrhage. The aim of this review is to consider mechanisms on the relationship between periodontitis and aneurysm rupture, focusing on recent evidence.

## Introduction

Recently, our group found the association with periodontitis, formation of intracranial aneurysms (IA), and the risk of aneurysmal subarachnoid hemorrhage (aSAH) [25]. Together with the prior reports that DNA of oral bacteria is found in the wall of many ruptured intracranial aneurysms [64, 65], these results raised the intriguing question whether periodontitis and oral bacteria participate to the formation of IAs and to their progression towards rupture. We review the current research about the association of oral infections with aneurysms and other vascular diseases, and discuss the potential mechanisms by which oral infections may modulate, contribute to, or even cause the degenerative remodeling of the aneurysm wall and as such predispose to the risk of aneurysmal subarachnoid hemorrhage.

### Intracranial aneurysms as a clinical challenge—need for identification of rupture-prone aneurysms

Intracranial aneurysms (IA) are pathological dilatations of cerebral arteries, most often saccular in shape and frequently found in proximal cerebral artery bifurcations [30]. Aneurysms are relatively common with a prevalence of 2–3% in the general population [70, 91].

Although unruptured IAs are common, intracranial hemorrhage caused by IA rupture is a quite rare event affecting 10–11/100000 population per year in Western populations [32]. In fact, based on epidemiological data, it seems that only approximately 1% of IAs rupture per year [70] and many of them actually never rupture during the lifetime of the person carrying them [41]. However, because of the serious consequences that IA rupture and subsequent intracranial hemorrhage have (40% mortality and most of the survivors being left with significant neurological impairment [54, 78]), many of the diagnosed unruptured IAs are treated to prevent rupture. Preventive treatment is done by endovascular embolization of aneurysm sack or by microsurgical ligation of the aneurysm neck, both of which exclude the aneurysm from systemic circulation. Both of the current treatment options are associated with significant risk of serious neurological complications (5–7%), including mortality (1–2%) [43, 53]. Therefore, it is paramount to focus intervention only to those IAs at risk of rupture. This remains a challenge.

The discrepancy between the relatively high prevalence of unruptured IAs and the low incidence of IA rupture, together with the observation that only 1/3 of unruptured IAs ruptured during a life-long follow-up [41], strongly suggests that IA formation and IA rupture are two separate phenomena with different pathological background. Moreover, these observations suggest that while some IA walls are able to adapt to the mechanical load imposed on them, in others degenerative remodeling makes the IAs rupture-prone. This in turn implies that by pharmacological manipulation of the adaptive or degenerative remodeling of the IA wall, it might be possible to keep the formed unruptured IAs stable and prevent rupture. To achieve this, the pathophysiological mechanisms guiding IA growth and rupture, i.e., adaptive and degenerative wall remodeling, need to be understood. Moreover, biomarkers for an IA wall capable of adaptive remodeling are needed to identify those IAs that can be stabilized with drug therapy.

### Intracranial aneurysms show chronic inflammation that is associated with wall degeneration and rupture

**Fig. 1:**
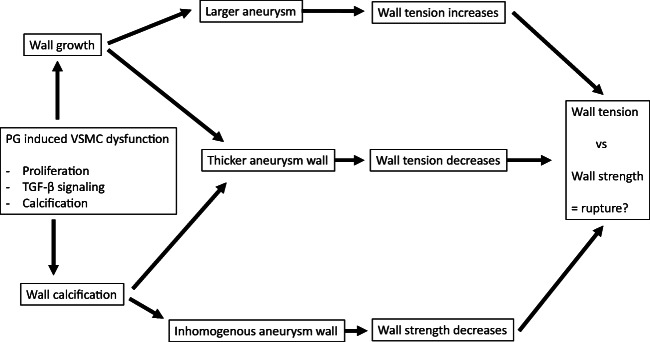
Circulating periodontal bacteria, their antibodies, circulating elastase and inflammatory reaction products causes endothelial dysfunction in arterial wall, which predisposes arterial wall to high flow condition. Simultaneously macrophage infiltration is observed in the arterial wall and IA formation accelerates

**Fig. 2:**
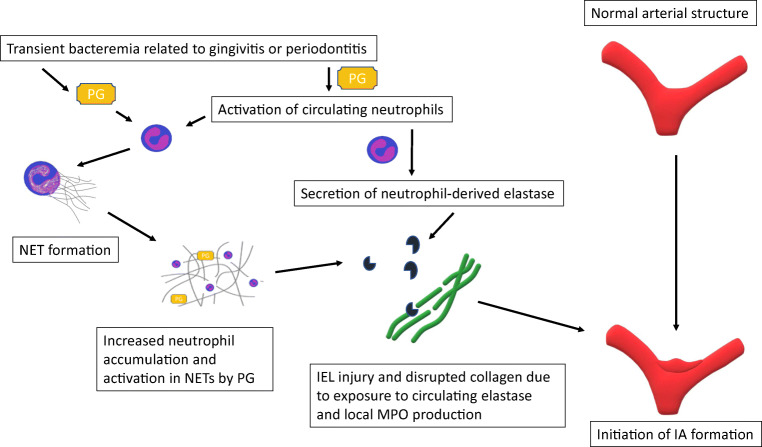
*Porphyromonas gingivalis* (PG) activates circulating neutrophils and induces formation of neutrophil extracellular traps (NET) in cerebral arteries. Endured PG challenge leads to neutrophil-derived elastase overload and MPO production causing disruption of the internal elastic lamina (IEL) of the arterial wall and IA formation initiates

Since human tissue samples from the site of IA initiation are inherently difficult to obtain, most of our knowledge of the pathophysiological mechanisms leading to IA formation is derived from animal models of induced IA formation [1, 27, 34, 39]. Current understanding is that IAs form as the end result of flow-driven inflammatory cell-mediated cerebral artery wall remodeling at the sites where high flow causes high wall shear stress (WSS) [22, 75](Fig. 1). However, not all cerebral artery bifurcations exposed to high WSS develop IAs, suggesting that additional factors are needed [22]. A common feature to IA initiation is the disruption of internal elastic lamina (IEL) [27, 39], resulting in distension of the vessel wall to the extent that collagen fibers allow [22]. In a mouse model of induced IA formation, IEL disruption is related to flow-induced macrophage infiltration [22, 34]. In humans, however, systemic elastase activity may play a significant role since increased serum elastase concentrations associate with IAs [10], although the source is unknown. Potential sources for circulating elastase are macrophages or neutrophils [13, 68]. Given that chewing or brushing teeth predisposes to transient bacteremia, especially in patients with gingivitis or periodontitis [16, 28, 49, 84], activation of circulating neutrophils as a response to transient bacteremia of periodontal pathogens with subsequent formation of neutrophil extracellular traps (NETs) and elastase release [13] could lead to wall degeneration (Fig. 2)—thus explaining why IA form for some but not all persons under high flow and shear stress towards bifurcations of cerebral arteries [75].

In humans, increased infiltration of leukocytes, mainly macrophages and to some extent T cells, is observed in ruptured IA walls compared with unruptured IA walls [18, 37, 55]. The observation that inflammatory cell infiltration is present in unruptured IAs as well as in ruptured IAs operated early after rupture, combined with the observation that this inflammatory cell infiltration does not associate with time from rupture [18], strongly implies that the inflammatory cell infiltration is present before rupture. Thus, inflammatory cell infiltration associates with wall degeneration in both unruptured and ruptured IA walls [56, 57]. In animal models of induced IA formation, blocking macrophage infiltration or activation inhibits the formation, growth, and rupture of the IAs [2, 3, 36, 74].

Inflammatory response mediated especially by macrophages is an essential part of the flow-induced vessel remodeling that leads to aneurysm initiation and growth [22]. However, in the human IA wall also a humoral immune response and activation of the complement system are observed and associating with wall degeneration in unruptured IA walls as well as with rupture [86, 87]. This strongly implies that the chronic inflammation seen in human IA walls is not just related to flow-induced remodeling, especially since there is very high variation in the inflammation degree among IAs [18, 55, 86] despite the fact that all are exposed to non-physiological flow. The cause of this inflammation in the IA wall is unclear. Potential triggers of inflammation include circulating lipids [20, 88] and periodontal and cariogenic bacteria [31, 64, 65].

### The highly prevalent oral infections, dental caries and periodontitis, and their systemic effects

Dental caries and periodontitis are both highly prevalent oral diseases worldwide despite the modern dental therapy and accumulating knowledge of oral diseases. Caries is an infective disease of hard tissues of the tooth, and the prevalence of untreated caries is one-third of the global population as well as in Europe [17] In the USA and Asia pacific, the prevalence varies between 22 and 25% [17]. Another common oral disease, periodontitis, is also to a large extent preventable inflammatory disease and highly underdiagnosed. Its severe form affects about 10% of the global population, and 7% in Asia pacific area [17]. In the USA, the prevalence of mild to moderate and severe periodontitis is reported to be 46% and 9% of adults, respectively [14]. In Europe, corresponding prevalence is 40–60% and 10–20% [17], respectively.

The microbial load on tooth surface, dental plaque, composed of hundreds of microbial species [59]. Periodontal pathogenesis initiates by microbial accumulation on the tooth surface resulting a reversible gingival inflammation known as gingivitis [77, 83], which may progress to periodontitis among susceptible individuals when the normally dominating beneficial species become lessened and replaced by pathogenic species [40]. Therefore, endured microbial burden, in form of dental plaque or calculus, leads to a chronic inflammatory disease—periodontitis. Without interventional therapy, periodontitis leads to breakdown of both soft and hard tissue mainly via inflammatory host response [11] causing loss of tooth attachment and eventually loss of alveolar bone in the jaws. Periodontitis not only degenerates tooth surrounding structures but affects the inflammatory system [35, 51, 52, 60, 62] and systemic health [12, 25, 38, 45, 46, 63, 79].

Periodontitis is associated with the risk of several diseases. For example, in rheumatoid arthritis, *Porphyromonas gingivalis* (PG), one of the most common periodontal pathogens, seems to be potential generator of autoantibodies [50]. Periodontitis has also been associated with atherosclerosis, stroke [38, 46, 63], and abdominal aortic aneurysms (AAA) [12, 45, 79]. Very recently, our group showed that periodontitis or gingivitis associate also with aSAH and IA formation [25]. In addition to this, another recent study reported an association between aSAH and being a carriage of specific strains of *Streptococcus mutans*, a significant contributor to tooth decay [31]. These clinical studies suggest that oral infections and exposure to oral pathogens associate with risk of IA formation and aSAH.

### Potential role of periodontal pathogens in the pathobiology of the intracranial aneurysm

In addition to the clinical association of periodontitis with IA formation and aSAH suggesting a potential causative association, a recent experimental study has reported that temporary ablation of the gut microbiome prevents IA formation through indirect modulation of the inflammatory remodeling in the cerebral arteries [21, 73]. This experimental study highlights the thus far unnoticed importance of microbial exposure in the pathogenesis of IAs.

While it has been long known that septic, bacterial emboli ending up in cerebral arteries may cause formation of so-called mycotic aneurysms through focal inflammation of the artery wall, most saccular IAs have been considered as being aseptic. However, a landmark study screening human IA wall tissue samples for the presence of any bacterial DNA demonstrated DNA of oral pathogens in up to 50% of ruptured IA walls [64, 65]. The fact that the remaining 50% were negative for any bacterial DNA demonstrates that IAs can form without direct bacterial involvement. The combination of having an epidemiological association between periodontal infection and IA formation and rupture [25, 66], experimental proof that microbiome exposure modulates IA formation [21, 73], and that even presence of oral pathogen remnants is found in many IA walls [64, 65] justifies the hypothesis that periodontal pathogens contribute to the risk of IA formation and rupture through indirect or direct modulation of the inflammatory remodeling in the cerebral artery and IA wall.

### Potential mechanisms through which oral pathogens can affect inflammatory remodeling in the IA wall

We will focus on those periodontal pathogens that have been identified in walls of ruptured intracranial aneurysms by Pyysalo et al. [64, 65] and discuss through their known role in other vascular diseases their potential contribution to IA pathogenesis.

The importance of macrophage infiltration for the inflammatory remodeling leading to IA formation has been demonstrated by several studies [3, 22, 36, 74, 80], including the landmark experimental studies by Kanematsu et al., Shimada et al., and Aoki et al. [3, 36, 74]. *P. gingivalis* can stay viable in human macrophages and dendritic cells [7, 76, 92] and thus possibly disseminate to IA wall via macrophage infiltration. *P. gingivalis* seems to be capable of modifying the function of dendritic cells, for example, the secretion of collagen degrading MMP-9 is promoted in dendritic cells that are infected with *P. gingivalis* [7]. In addition, presence of *P. gingivalis* and *Fusobacterium nucleatum* (FN) DNA has shown to induce proinflammatory cytokine production in macrophages [71, 72]. Thus *P. gingivalis* infection of the macrophages that infiltrate the cerebral arteries would likely lead to excessive collagen degradation and local inflammation response, predisposing to IA formation.

**Fig. 3:**
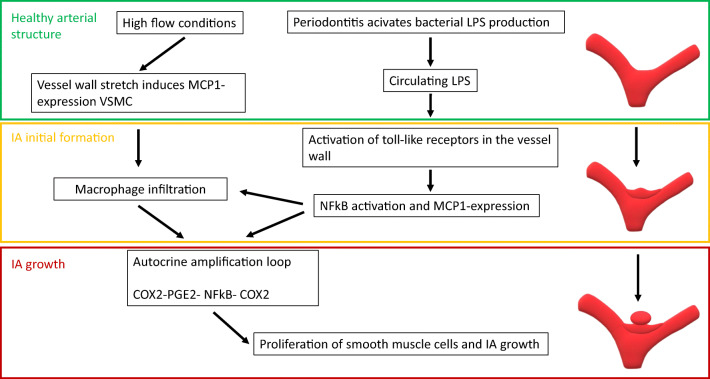
High flow conditions stretch the vessel wall and induce monocyte chemoattractant protein 1 (MCP-1) in vascular smooth muscle cells (VSMC) attracting invading macrophages to vessel wall. Similar MCP-1 production is caused by lipopolysaccharide (LPS) production of periodontal bacteria via activating nuclear factor kB (NFkB) -signaling via toll-like receptors. These mechanisms lead to cyclo-oxygenase 2-prostaglandin E2-NFkB-cyclo-oxygenase 2 (COX2-PGE2-NFkB-COX2)-amplifying loop in IA wall and lead to proliferation of smooth muscle cells expanding the IA

Another mechanism through which circulating components of oral bacteria could enhance or amplify the inflammatory remodeling of the cerebral artery wall is through activation of toll-like receptors by LPS, e.g., from *P. gingivalis* in luminal thrombus [12](Fig. 3). Toll-like receptors are expressed in aneurysm walls [64] and their stimulation triggers activation of the transcription factor NFkB [3], a main mediator of the inflammatory remodeling leading to IA formation [3]. As a result, having oral bacteria-derived components in the circulation would promote inflammatory vessel remodeling triggered by, e.g., high flow, and thus predispose to IA formation.

**Fig. 4:**
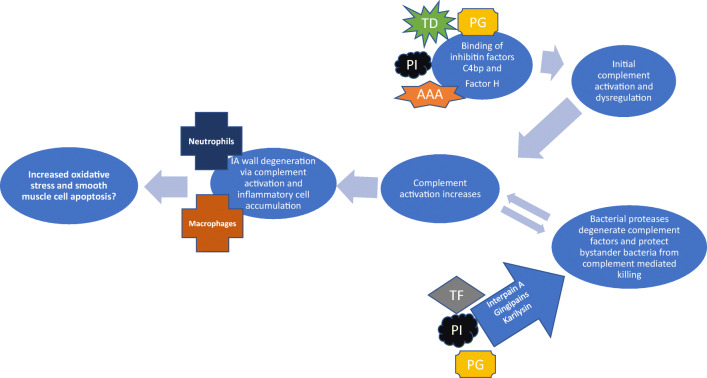
Periodontal bacteria have ability to dysregulate complement activity with their proteinase production (*Porphyromonas gingivalis* PG, *Prevotella intermedia* PI, and *Tannerella forsythia* TF) or directly binding complement factor C4bp and factor H (*Porphyromonas gingivalis* PG, *Prevotella intermedia* PI, *Treponema denticola* TD, and *Aggregatibacter actinomycetemcomitans* AAA). These bacteria simultaneously accelerate complement activity yet evade complement-mediated killing is unique and could partially explain inflammation in IA wall, especially since oral bacterial DNA is found in unruptured and ruptured IA walls

Viable periodontal bacteria, namely *P. gingivalis*, *Aggregatibacter actinomycetemcomitans* (AA), and *Treponema denticola* (TD), have been found in human atheromas [8, 44, 67] (Fig. 4). In vitro and in vivo studies have well established that it is biologically plausible that periodontitis accelerates atherosclerosis [6, 33, 48, 69]. Recent mechanistic study using ApoE^null^ mouse model revealed an ability of *P. gingivalis* to actively invade luminal side of aortic wall, retaining its vitality and leading to greater aortic plaque area [47, 90]. In another mechanistic study using a pig model, circulating *P. gingivalis* promoted atherosclerosis not only in hypercholesterolemic pigs but also normocholesterolemic ones [6]. These findings suggest that the active invasion of periodontal pathogens in the artery wall with or without presence of cholesterol can be a potential trigger of atherosclerotic activity. Nearly all intracranial aneurysms feature at least minor atherosclerotic changes and advanced atherosclerotic changes are seen in large numbers [20, 42]. Of note is that the accumulation of lipid and oxidized low-density lipoprotein (oxLDL) was found to associate with IA wall degeneration, loss of mural cells, and also IA rupture, despite normal lipid levels [20]. Interestingly, not only has *P. gingivalis* been shown to accelerate atherosclerosis [6, 90] but immunization against it has been shown to reduce atherosclerotic changes [24, 89]. Thus, accumulation of oxidized lipids to the IA wall and subsequent atherosclerotic remodeling might be altered indirectly by systemic immunization induced by local periodontitis. It seems possible that the presence of *P. gingivalis* in the IA wall or immune response induced by *P. gingivalis* elsewhere in the body may modulate directly or indirectly the progression of atherosclerotic changes and lipid-induced inflammation in the IA wall.

Periodontitis is common also among patients with abdominal aortic aneurysms (AAA) and periodontal bacterial DNA have been identified in AAA wall structure as well as in the thrombus lining the AAA inner wall [12, 45]. Presence of periodontal bacteria promotes AAA growth, i.e., enlargement of luminal diameter [4, 12], and thus could predispose AAA to rupture. *P. gingivalis* appears to promote AAA pathogenesis by maintaining inflammation and increasing oxidative stress in the AAA wall via adhesion to the intraluminal thrombus [12]. *P. gingivalis* has an affinity for thrombus and can passively accumulate to sites of thrombosis if it is present in the bloodstream [12]. In the thrombus, *P. gingivalis* is capable of inducing the formation of neutrophil-derived extracellular traps (NETs), which increases further the accumulation of neutrophils to the thrombus [12]. Fontaine et al. have shown that the neutrophils in the intraluminal thrombus are a major source of proteolytic activity as they release matrix metalloproteinases MMP-8, MMP-9, and elastase [15, 29]. Furthermore, the neutrophils attracted to luminal thrombus produce proteases and cytotoxic enzymes such as myeloperoxidase (MPO) [15, 29], which generates, e.g., hydrogen peroxide and hypoclorous acid intended to kill bacteria and leading to very high oxidative stress locally [26]. In addition to increasing the local recruitment of neutrophils, *P. gingivalis* can also activate the neutrophils in the thrombus, thus accelerating the neutrophil-derived proteolytic and cytotoxic injury [12](Fig. 2). This cytotoxic injury is in part mediated by MPO. In summary, presence of *P. gingivalis* in the intraluminal thrombus or adjacent AAA wall can trigger or enhance the activation and recruitment of neutrophils, leading to excessive proteolytic and cytotoxic injury to the vessel wall. Similarly to AAAs, neutrophils are recruited to the luminal thrombus lining the IA walls [18, 19, 56]. Luminal thrombus is common in IAs [18], as is periodontitis in IA patients [25, 66] suggesting that *P. gingivalis* might accelerate neutrophil-driven thrombus-derived proteolytic and cytotoxic injury in the IA wall similarly to the AAA walls. Indeed, MPO expression has been demonstrated in human IA walls [23, 56] and locally higher MPO concentrations have been observed in serum from aneurysm sac than to femoral artery blood [9].

Besides the infiltration of inflammatory cells such as macrophages and neutrophils, activation of the complement system is involved in IA wall degeneration and rupture [86, 87]. It has been shown earlier that periodontal bacteria can dysregulate complement activity in vitro by binding to or degenerating complement factors [35, 51, 52, 60, 62]. Of periodontal pathogens, specific strains of *Prevotella intermedia* (PI) and *P. gingivalis* participate in complement activation by binding factor H [51] and C4bp respectively [61]. *Treponema denticola* (TD) and *A. actinomycetemcomitans*, common periodontal pathogens, have ability to bind factor H [5, 52]. These bacteria do not only dysregulate complement activity but also evade complement-mediated killing in response of binding factor H [51, 52]. It is noteworthy that DNA of both *P. intermedia* and *T. denticola* have been identified in some of ruptured IAs [64, 65]. Interestingly, *P. gingivalis*, *P. intermedia*, and *Tannerella forsythia* (TF) are capable of evading complement-mediated killing by altering complement activity and degenerating complement factors via their bacterial proteases, while protecting other bystander-bacteria [35, 60, 62]. This ability of periodontal bacteria to activate complement while evading complement-mediated killing may in part explain the complement activation seen in the IA wall in association with IA wall degeneration.

In addition to the mechanisms described above, periodontal bacteria may also trigger IA wall degeneration via endothelial dysfunction, which seems to be one of the key events that triggers the lipid accumulation, luminal thrombosis, and inflammation that associate with the IA wall degeneration [19]. A recent meta-analysis concluded that periodontal bacteria predispose to endothelial dysfunction [58]. Moreover, endothelial dysfunction improves with periodontal treatment [58, 85]. Endothelial dysfunction can be at least in part caused by bacterial burden of periodontitis and furthermore, endothelial dysfunction could explain why periodontal bacteria are often found in the sites of vascular diseases, e.g., IA walls and atheromas. Interestingly, periodontitis patients also have higher concentration of circulating oxidized LDL particles in the blood [81, 82], another mechanism more through which periodontitis can accelerate local atherosclerotic remodeling in the IA wall.

## Conclusion

Although the association of aneurysm rupture with chronic inflammation of the aneurysm wall is well established, the triggers for this inflammatory reaction are incompletely understood. The recent finding that periodontitis or gingivitis associates with increased risk of aSAH together with DNA of oral bacteria found in the walls of many IAs raises the hypothesis that oral infections are related to the inflammation of the IA wall. In this review, we presented several mechanisms by which periodontitis and periodontal pathogens can contribute to the degenerative remodeling of the IA wall. These mechanisms merit further investigation and may reveal a previously unknown and treatable risk factor for the deadly aneurysmal subarachnoid hemorrhage.
